# Light-driven directional ion transport for enhanced osmotic energy harvesting

**DOI:** 10.1093/nsr/nwaa231

**Published:** 2020-09-08

**Authors:** Kai Xiao, Paolo Giusto, Fengxiang Chen, Ruotian Chen, Tobias Heil, Shaowen Cao, Lu Chen, Fengtao Fan, Lei Jiang

**Affiliations:** Max Planck Institute of Colloids and Interfaces, Department of Colloid Chemistry, Potsdam D-14476, Germany; Max Planck Institute of Colloids and Interfaces, Department of Colloid Chemistry, Potsdam D-14476, Germany; Key Laboratory of Bio-inspired Smart Interfacial Science and Technology of Ministry of Education, School of Chemistry, Beihang University, Beijing 100191, China; State Key Laboratory of New Textile Materials and Advanced Processing Technologies, Wuhan Textile University, Wuhan 430200, China; State Key Laboratory of Catalysis, 2011-iChEM, Dalian National Laboratory for Clean Energy (DNL), Dalian Institute of Chemical Physics (DICP), Chinese Academy of Sciences, Dalian 116023, China; Max Planck Institute of Colloids and Interfaces, Department of Colloid Chemistry, Potsdam D-14476, Germany; Max Planck Institute of Colloids and Interfaces, Department of Colloid Chemistry, Potsdam D-14476, Germany; State Key Laboratory of Advanced Technology for Materials Synthesis and Processing, Wuhan University of Technology, Wuhan 430070, China; Max Planck Institute of Colloids and Interfaces, Department of Colloid Chemistry, Potsdam D-14476, Germany; Key Laboratory of Bio-inspired Smart Interfacial Science and Technology of Ministry of Education, School of Chemistry, Beihang University, Beijing 100191, China; State Key Laboratory of Catalysis, 2011-iChEM, Dalian National Laboratory for Clean Energy (DNL), Dalian Institute of Chemical Physics (DICP), Chinese Academy of Sciences, Dalian 116023, China; Key Laboratory of Bio-inspired Smart Interfacial Science and Technology of Ministry of Education, School of Chemistry, Beihang University, Beijing 100191, China

**Keywords:** ion pump, ion transport, nanofluidic, porous membrane, carbon nitride

## Abstract

Light-driven ion (proton) transport is a crucial process both for photosynthesis of green plants and solar energy harvesting of some archaea. Here, we describe use of a TiO_2_/C_3_N_4_ semiconductor heterojunction nanotube membrane to realize similar light-driven directional ion transport performance to that of biological systems. This heterojunction system can be fabricated by two simple deposition steps. Under unilateral illumination, the TiO_2_/C_3_N_4_ heterojunction nanotube membrane can generate a photocurrent of about 9 μA/cm^2^, corresponding to a pumping stream of ∼5500 ions per second per nanotube. By changing the position of TiO_2_ and C_3_N_4_, a reverse equivalent ionic current can also be realized. Directional transport of photogenerated electrons and holes results in a transmembrane potential, which is the basis of the light-driven ion transport phenomenon. As a proof of concept, we also show that this system can be used for enhanced osmotic energy generation. The artificial light-driven ion transport system proposed here offers a further step forward on the roadmap for development of ionic photoelectric conversion and integration into other applications, for example water desalination.

## Teaser

Light-driven directional ion transport that is inspired by light-driven ion pump was reported to boost osmotic energy harvesting.

## INTRODUCTION

Nature's biochemical machinery is a source of inspiration for development of artificial molecular devices or ion transport systems designed to emulate the form and function of their biological counterparts. A typical example is that the cellular metabolism in living organisms depends on the compartmentalization of ions, small molecules and macromolecules being maintained and managed by means of transmembrane active transport. Inspired by this, artificial systems have been developed that are capable of transporting molecules against concentration gradients, such as molecular motors [1,2] and molecular pumps [3]. Photosynthesis by green plants or archebacter is the most important biochemical process in nature, providing most of the energy we need and a comfortable environment suitable for biological survival. With the development of modern science, photosynthesis-inspired light-driven physical and chemical processes have attracted extraordinary attention. We mention, for instance, photocatalytic chemical reactions [4] or the photovoltaic cell [5], both of which occupy thousands of research groups globally and have already created tremendous economic value. However, artificial photosynthesis involving complex physical and chemical processes is still one of the most tough, but also promising, missions in the field of bionics [6–9].

In terms of energy harvesting, artificial light-driven ion transport is very attractive because the different photosynthetic processes of both green plants and halobacteria involve a step during which protons/ions are pumped from low concentration to high concentration to create an electrochemical potential, which is then used for ATPase to produce ATP. Consequently, the realization of artificial light-driven ion transport is the key point for a new ‘ionic’ mode of solar energy harvesting and storage [10].

**Figure 1.**
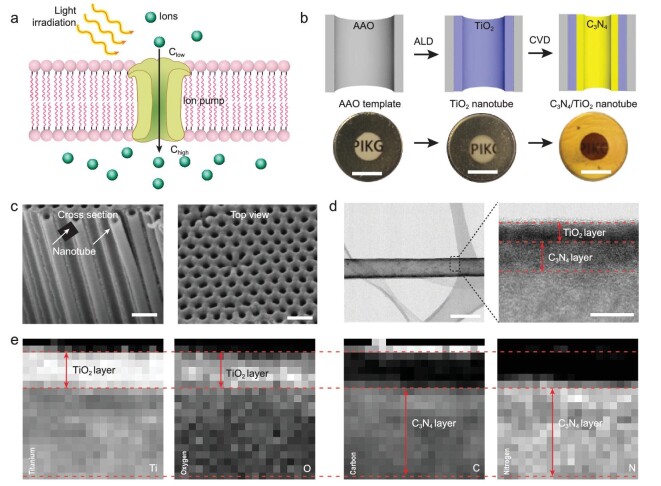
Fabrication process and characterization of TiO_2_/C_3_N_4_ semiconductor heterojunction nanotubes. (a) Simplified schematic of light-driven ion transport system. (b) Fabrication process of TiO_2_/C_3_N_4_ heterojunction nanotubes including two steps. Step 1: TiO_2_ layer deposition by ALD; Step 2: C_3_N_4_ layer deposition by CVD (scale bar, 0.5 cm). (c) SEM images of TiO_2_/C_3_N_4_ heterojunction nanotube membrane from cross section and top view (scale bar, 200 nm). (d) TEM image of signal TiO_2_/C_3_N_4_ nanotube (scale bar, 100 nm) and enlarged wall surface (scale bar, 10 nm). (e) Elemental maps of TiO_2_/C_3_N_4_ heterojunction nanotube wall. Each pixel covers 1 nm^2^.

Remarkable progress has also been achieved toward realization of permselectivity of protons or alkaline earth metal ions across membranes by light-induced charge separation [11–13], photo-isomerization [14,15] and solid-state nanochannels [16,17]. This ability raises a pertinent question for artificial light-driven ion transport systems: is there a way to drive ion transport by light in an easy and universal manner, and then realize an efficient ‘ionic’ energy harvesting as in nature? Herein, we report development of an artificial light-driven ion transport system via a semiconductor heterojunction nanotube membrane that drives ion transport in a specific direction under unidirectional illumination for photocurrent generation (Fig. 1a). We demonstrate that such semiconductor heterojunction nanotubes consisting of titanium oxide (TiO_2_) and polymeric carbon nitride (C_3_N_4_) enable efficient light-driven ion transport and tunable ion transport direction by controlling the heterojunction structure.

## RESULTS AND DISCUSSION

### Fabrication and characterization of TiO_2_/C_3_N_4_ heterojunction nanotubes

The heterojunction of the semiconductor nanotubes used here is a TiO_2_/C_3_N_4_ heterojunction, which was fabricated by two deposition steps (Fig. 1b and Supplementary Fig. 1). In the first step, TiO_2_ nanotubes with various wall thicknesses were fabricated by an atom layer deposition (ALD) method using porous anodic aluminum oxide (AAO) membrane with pore diameter about 100 nm as the substrate (Supplementary Fig. 2). Then, the amorphous TiO_2_ nanotubes were crystallized by thermal annealing at 500°C for 2 hours. In the second step, the anatase TiO_2_ nanotubes (Supplementary Fig. 3) were coated with a 10 nm layer of C_3_N_4_ (Supplementary Fig. 4) by chemical vapor deposition (CVD) [18]. In this way, a TiO_2_/C_3_N_4_ heterojunction nanotube was fabricated (Fig. 1c). For analytical reasons, the carbon nitride nanotube can be released by chemical etching of the AAO substrate by 5 wt% phosphoric acid. Figure 1d shows a typical TEM image of a TiO_2_/C_3_N_4_ heterojunction nanotube, with the enlarged wall section showing that the wall is composed of an inner TiO_2_ layer and outer C_3_N_4_ layer. High-resolution EDX measurements of the partial wall section in Fig. 1e and Supplementary Fig. 5 show that the inner TiO_2_ layer is about 5 nm thick, while the outer C_3_N_4_ layer is about 10 nm thick (The pixel intensity represents the concentration of the related elements, respectively. Each pixel covers 1 nm^2^). This is a representative of samples used in this work, but the thickness of the C_3_N_4_ and TiO_2_ layers can be well controlled (see below). The length of all samples is the same, about 60 μm. The obtained heterojunction nanotubes were investigated by X-ray diffraction (XRD) measurements, FT-IR spectroscopy and X-ray photoelectron spectroscopy (XPS) (Supplementary Figs 6–8). All of the results indicate formation of TiO_2_/C_3_N_4_ heterojunction nanotubes.

### Light-driven ion transport phenomenon

**Figure 2.**
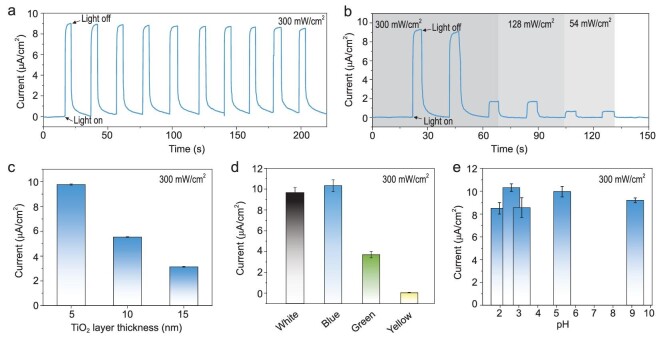
Light-driven ion transport performance of TiO_2_/C_3_N_4_ semiconductor heterojunction nanotube membrane. (a) Measured cyclic constant zero-volt current with alternating illumination at 0.1 M KCl concentration. (b) Zero-volt current as a function of light density of 54 mW/cm^2^, 128 mW/cm^2^ and 300 mW/cm^2^. (c) Zero-volt current as a function of TiO_2_ layer thickness. (d) Zero-volt current as a function of monochromatic light (blue: 405 nm; green: 515 nm; yellow: 590 nm) with same power density of 300 mW/cm^2^. The ionic current is consistent with the light absorbance of outer C_3_N_4_ layer. (e) Zero-volt current as a function of pH. Error bars in (c–e) represent the standard deviations of five independent experiments.

The light-driven ion transport properties were measured using a home-made electrolyte cell, as we have reported previously [19,20]. The TiO_2_/C_3_N_4_ heterojunction nanotube membrane was symmetrically placed in contact with a 0.1 M KCl solutions and initially illuminated from one side. Figure 2a shows the cycle-constant zero-volt current across the nanotube membrane by simulated solar illumination of 300 mW/cm^2^. Without illumination, the zero-volt current is almost zero, while it increased to about 9 μA/cm^2^ with illumination, indicating that light provides an external force to drive ions to move. By calculation, the changed current translates into the fact that a single nanotube actively transports ∼5500 ions per second, an unprecedented breakthrough for artificial light-driven ion transport systems and much closer to values for the bacteriorhodopsin sodium pump [21] or halorhodopsin Cl ion pump [22]. The directional photo-driven ion transport phenomenon can be directly confirmed by the change of ion concentration in the two cells (Supplementary Fig. 9), which can be monitored in real time with a scanning ion-selective electrode technique (SIET). In addition, the membrane shows an instantaneous stable and fully repeatable response to illumination. The ionic current is still stable even at longer illumination (Supplementary Fig. 10). Further measurements show that the ion transport is closely connected to the illumination power density. The dependence can be confirmed by the ionic current shown in Fig. 2b. With decrease of power density from 300 mW/cm^2^ to 54 mW/cm^2^, the ionic current decreases gradually from 9 μA/cm^2^ to 0.8 μA/cm^2^. It is worth mentioning that the photo-induced voltage is also positively correlated with light power density (Supplementary Fig. 9), while it is only dozens of millivolts and much smaller than that for pure C_3_N_4_ nanotube membrane [20].

The wall thickness of TiO_2_ nanotube has an obvious effect on the light-driven ion transport properties (Fig. 2c). With increase of wall thickness from 5 nm to 15 nm (Supplementary Fig. 2), the ionic current decreases from about 9 μA/cm^2^ to 2.5 μA/cm^2^. This could be ascribed to a less efficient photochemical charge separation and more interfacial recombination of electrons and holes in thicker case [23]. The light-driven ion transport system also shows an obvious relationship with the light wavelength (Fig. 2d). When applying various monochromatic light with the same power density (300 mW/cm^2^), white light and high-energy blue light have a comparable high ‘power’ to drive ion transport, while low-energy green and yellow light show a much weaker effect (Supplementary Fig. 11). This is consistent with the light absorbance of the exposed C_3_N_4_ layer (Supplementary Fig. 12) [24]. In general, the isoelectric point of C_3_N_4_ fabricated by different precursors is in the range of 3.5 to 5 [25], while the light-driven ion transport system is universal and works constantly in a wider pH value range from 1.9 to 9.5 (Fig. 2e). In strong alkali solution with a pH value of 12.5 it shows a different phenomenon as C_3_N_4_ is surface-hydrolyzed under such conditions (Supplementary Fig. 13) [26,27]. Meanwhile, the ionic current shows a positive correlation with electrolyte concentration (Supplementary Fig. 14).

### Mechanism of light-driven ion transport

**Figure 3.**
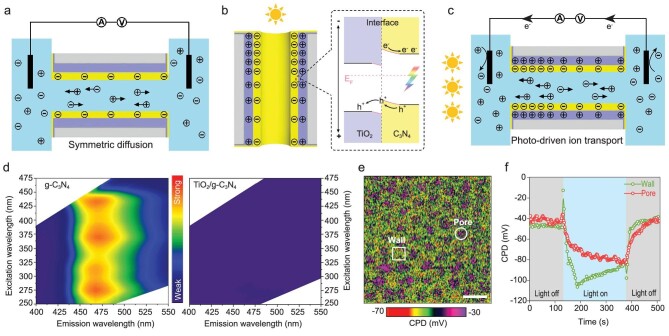
Mechanism of light-driven ion transport phenomenon. (a) Schematic of the surface charge distribution on the nanotube before illumination, in which condition low density negative charge is homogeneously distributed over the nanotube. (b) Light-induced separation of electrons and holes of C_3_N_4_. The holes transfer from C_3_N_4_ to TiO_2_. (c) Schematic of the surface charge distribution on the nanotube after unilateral illumination, in which condition the separation of electrons and holes results in heterogeneous negative charge distribution. (d) Fluorescent mapping of C_3_N_4_ (left) and TiO_2_/C_3_N_4_ (right) nanotube membranes. (e) KPFM image of the TiO_2_/C_3_N_4_ nanotube membranes. Scale bar, 200 nm. (f) Surface potential (CPD) evolution with light on and off in pore area and wall area of the TiO_2_/C_3_N_4_ nanotube.

The surface charge redistribution of the heterojunction nanotube resulting from photo-induced separation of electrons and holes is thought to be key

to the light-driven ion transport phenomenon. As illustrated in Fig. 3a, the initial TiO_2_/C_3_N_4_ nanotube has a symmetric weakly negative charge because of the acidity of the inner C_3_N_4_ layer [28]. In this condition, there is no ionic current in the external circuit. When illuminated from one side of the H-cell, the surface charge density on the irradiated side of the TiO_2_/C_3_N_4_ nanotube increases because the built-in potential in the heterojunction resulting from band bending will drive photogenerated holes to move from the C_3_N_4_ layer to the TiO_2_ layer (Fig. 3b). This results in a positively charged TiO_2_ layer and a negatively charged C_3_N_4_ layer. As the asymmetric negative surface charge is created, cations will move from the non-illuminated side to the illuminated side, while anions move oppositely (Fig. 3c). In this way, a light-driven ion transport system develops. Previous work has already shown that a single phase C_3_N_4_ nanotube exhibits similar, but weaker, light-driven ion transport properties [20,29], caused by less efficient photocharge separation [30,31]. In the present system, the nanoscopic TiO_2_/C_3_N_4_ heterojunction structure provides two different phases for each charge. The proposed mechanism is further confirmed by fluorescent mapping. As shown in Fig. 3d, the fluorescent mapping of the C_3_N_4_ nanotube system (left) exhibited fluorescence signals, which represent recombination of photo-generated electrons and holes; whereas for the TiO_2_/C_3_N_4_ heterojunction system (right), the fluorescence is negligible (Supplementary Fig. 15), which means that radiative recombination is effectively suppressed in the heterojunction structure.

To directly observe the charge distribution, we mapped the surface potential by Kelvin probe force microscopy (KPFM) under unilateral illumination. For the C_3_N_4_ nanotube, the potential of the pore area is 40 mV lower than that of the wall area, indicating upward band bending and electron capture by the C_3_N_4_ nanotube surface. Meanwhile, light irradiation increased the surface potential in both areas, indicating the n-type semiconductor property of C_3_N_4_ (Supplementary Fig. 16). As for the TiO_2_/C_3_N_4_ heterojunction structure, the surface potential of the pore area exceeds that of the wall area by 10 mV (Fig. 3e and Supplementary Fig. 17), indicating band bending and generation of built-in electrical field in the heterojunction. After light irradiation, the surface potentials of both the pore area and the wall area decreased, giving direct evidence for directional transport of photogenerated electrons (to the outer C_3_N_4_ layer) and holes (to the inner TiO_2_ layer) (Fig. 3f). The observed electron accumulation at the illuminated side both on the wall and in the pore coincides with the measured cation

migration towards the illuminated surface (Fig. 3c), thus indicating that the light-induced charge redistribution is responsible for the driving force of ion migration [32].

### Reverse ion transport by C_3_N_4_/TiO_2_ heterojunction nanotubes

**Figure 4.**
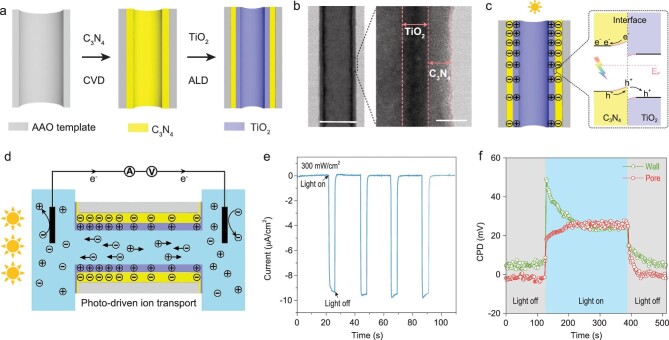
Fabrication of C_3_N_4_/TiO_2_ heterojunction nanotubes and their performance. (a) Schematic fabrication process of the C_3_N_4_/TiO_2_ heterojunction nanotube. Step 1: C_3_N_4_ layer deposition by CVD; Step 2: TiO_2_ layer deposition by ALD. (b) TEM image of signal C_3_N_4_/TiO_2_ nanotube (scale bar, 100 nm) and enlarged wall surface (scale bar, 10 nm). (c) Light-induced separation of electrons and holes of C_3_N_4_. The holes will transfer from C_3_N_4_ to TiO_2_. (d) Schematic of surface charge distribution and ion transport in the nanotube after unilateral illumination. (e) Measured cyclic constant zero-volt current with alternating illumination. (f) CPD evolution with light on and off in pore area and wall area of C_3_N_4_/TiO_2_ nanotube.

The light-driven ion transport can also be easily reversed by changing the position of C_3_N_4_ and TiO_2_. We fabricated a second C_3_N_4_/TiO_2_ nanotube membrane by the same deposition methods but with reverse order (Fig. 4a). Now, C_3_N_4_ is placed in the inner layer and TiO_2_ in the outer layer. Figure 4b shows a typical TEM image of the C_3_N_4_/TiO_2_ heterojunction nanotube and the enlarged wall section shows clearly that the wall is composited by an inner C_3_N_4_ layer and an outer TiO_2_ layer, both of which have a thickness of about 7 nm (a representative sample). With the unilateral illumination, the outer TiO_2_ layer should be positively charged from the directional movement of photogenerated holes (Fig. 4c). In this way, the anions will move from the non-illuminated side to the illuminated side (Fig. 4d). Figure 4e shows the cyclic constant zero-volt current across the nanotube membrane under a simulated solar illumination of 300 mW/cm^2^. Without illumination, the zero-volt current is almost zero, while it increased to about −9 μA/cm^2^ under illumination, which indicates that light provides an opposite external force for ion pumping. Figure 4f shows clearly that the reverse C_3_N_4_/TiO_2_ combination yields an opposite transmembrane photovoltage compared to the TiO_2_/C_3_N_4_ combination (Fig. 3f), in agreement with the reversed ion transport performance and further confirming the mechanism of light-driven ion transport. We can assume that not only can the ion transport direction be directly controlled using different semiconductor heterojunction combinations, but also the photocurrent value can be adjusted by combining different semiconductors with suitable band gaps. This suggests that semiconductor heterojunction nanotubes are a universal way to realize light-driven ion transport.

### Photo-enhanced blue energy generation

**Figure 5.**
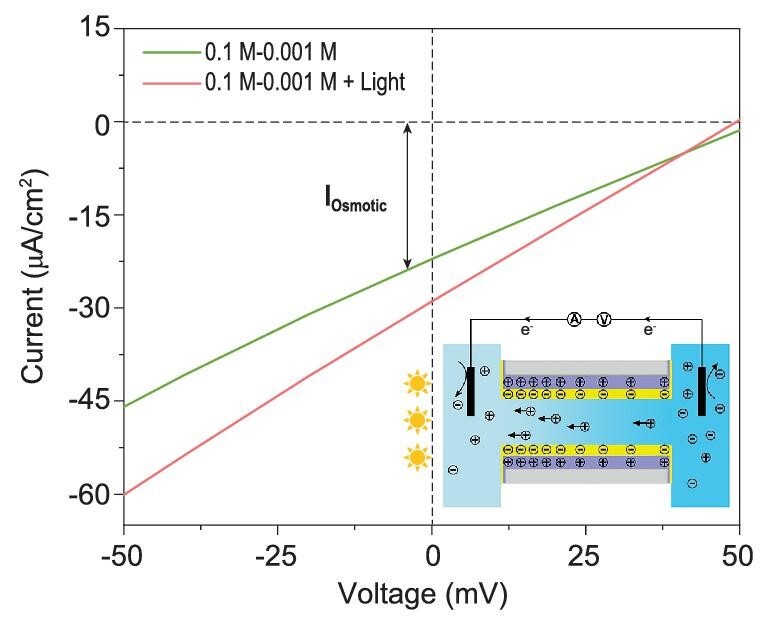
Potential application of photo-driven ion transport for enhanced osmotic energy generation. The typical current–voltage curves before and after light (300 mW/cm^2^) irradiation at 100-fold (C_H_ = 0.1 M; C_L_ = 0.001 M) KCl concentration gradient.

Beyond providing a novel method for photoelectrical energy conversion, this ion transport system can be potentially integrated into other energy harvesting approaches, for example to harvest salinity gradient energy, also called ‘blue energy’ [33–35]. Blue energy is a sustainable, abundant and inexpensive source of clean energy that is mainly stored in the sheer amount of available fresh and salty water being mixed (e.g. at the Yangtze river muzzle) [36]. The recently developed nanofluidic reverse-electrodialysis (NRED) method provides a very appealing way to harvest this energy and works again by a charged nanochannel or porous nanochannel membranes [10,37]. In the NRED process, surface charge density plays a crucial role. Generally speaking, high surface charge density will boost the blue energy power density. The weakly charged TiO_2_/C_3_N_4_ heterojunction nanotube membrane already can be used to harvest blue energy, but creates an osmotic current of only 21 μA/cm^2^ employing a 100-fold (0.1 M-0.001 M) concentration gradient (Fig. 5). With 300 mW/cm^2^ light irradiation from the low concentration side, the osmotic current increased to about 28 μA/cm^2^. This enhanced osmotic current is ascribed to the increased surface charge density induced by light irradiation, but also to the pumping flux being of the order of the current increase. The present light-driven ion transport system thereby provides an opportunity to integrate solar energy and salinity gradient energy [32], potentially overcoming the disadvantages of low energy efficiency and poor power density currently associated with blue energy.

## CONCLUSION

In summary, we report that semiconductor heterojunction nanotubes, herein using TiO_2_/C_3_N_4_ heterojunction nanotubes as an example, can be used for constructing an artificial light-driven ion transport system, which can then be used for ionic energy generation. The light-driven ionic current can reach up to about 9 μA/cm^2^, three times that of C_3_N_4_ nanotubes. The ionic transport direction can easily be reversed by modifying the semiconductor deposition sequence. The enhanced and flexible light-driven ionic current can be ascribed to redistribution of surface charge across nanotubes. In addition, we may expect that other semiconductor or semiconductor heterojunction nanostructures, which are currently used for photocurrent generation, for example two-dimensional van der Waals semiconductors [38] or 2D MOF [39], will exhibit similar light-driven ion transport performance. Most importantly, not only ions but also specific charged organic molecules up to small peptides are expected to be transported with the aid of this novel approach for directed molecular movement.

## Supplementary Material

nwaa231_Supplemental_File
