# Supplementary material for: Light-driven directional ion transport for enhanced osmotic energy harvesting
Source: Natl Sci Rev. 2020 Sep 8;8(8):nwaa231. doi: 10.1093/nsr/nwaa231 (PMC8363323; doi:10.1093/nsr/nwaa231)
Supplement: nwaa231_Supplemental_File [file nwaa231_supplemental_file.docx]

**Supporting information for**

**Light-driven directional ion transport for enhanced osmotic energy harvesting**

**Sample preparation.** TiO_2_ on the surface of AAO was carried out in a self-made hot-wall closed ALD reactor equipped with gas-flow system. Nitrogen (N_2_) with a purity of 99.99% was used as both purging and carrying gas at a steady flow rate of 50 sccm throughout the ALD process. Titanium (IV) isopropoxide (TIP) and deionized water were used as precursors for the TiO_2_ coating. Before the ALD process, the treated AAO were placed in the ALD reactor and kept at 150 °C for 30 min in vacuum (~0.5 Torr) to reach an equilibrium. Then, the TIP and H_2_O were alternatingly introduced into the ALD reactor. To produce adequate vapor pressures and prevent condensation of liquid, the TIP was heated to 80 °C, while water was maintained at room temperature. One complete round of the ALD cycle consisted of pulse (TIP)/ exposure (TIP)/purge (N_2_)/pulse (H_2_O)/ exposure (H_2_O) /purge (N_2_), corresponding to 0.02 s/8 s/25 s/0.1 s/8 s/25 s, respectively. By controlling the cycle numbers, the precise thickness of TiO_2_ layers could be achieved. Figure S 1b shows the temperature and time set for the fabrication of TiO_2_ layers.

The formation mechanism of the C_3_N_4_ layer was obtained from thermal condensation of melamine by a planarGROW-3S-OS CVD System, for organic semiconductor, provided by planarTECH, as previously reported elsewhere.^1,2^ In the present case, after the melamine was completely sublimed at 300°C, the substrate, i.e. AAO or AAO coated with TiO_2_ layer, was further kept at 550°C for additional 150 min at a pressure of 10 Torr. The samples were let cool down naturally and collected at room temperature. Figure S1a shows the temperature set in accordance with the time variation in two tube furnaces of CVD. In the whole process, nitrogen is used to protect the system from oxygen and carry precursor vapor from the upstream of the tube during the reaction. After the polymerization process, the furnace was left to cool down to room temperature.

**Electrochemical measurement.** The light-driven ion transport properties were studied by measuring the zero-volt ionic current and open circuit voltage through the nanotube membranes with and without light illumination. A membrane was mounted between two chambers of the self-made cells, which are full of electrolytes. To avoid light, a protected Ag/AgCl electrode was used to collect the current and voltage signals. Ionic current (photocurrent) was measured by a Keithley 6430 picoammeter (Keithley Instruments, Cleveland, OH). To the light density dependent measurements, the light density can be controlled by adjusting the distance between light source and membrane, and measured using a power meter. Four different light sources (while, blue, green, and yellow) with the same power density (300 mW/cm^2^) were used in this work. The light irradiation time was controlled to 5s to avoid photo-thermal effect.

**KPFM measurement.** The KPFM measurements were carried out under an ambient atmosphere on the basis of a commercial AFM system (Bruker Dimension Icon). Pt/Ir-coated Si tips with spring constant of 1-5 N m^–1^ and resonant frequency of 60-100 kHz (Bruker SCM-PIT) were used for the measurements. The KPFM maps the surface potential (contact potential difference (CPD)) of the samples with energy resolution of ~ 5 mV in an amplitude-modulated (AM)-KPFM mode with a.c. voltage of 0.5 V. The lift mode was adopted with a low lift height of 20 nm to minimize the possible cross-talk effect by the compensation of the tip and cantilever. A 405-nm laser was used to excite the top side of the sample with a light intensity of ~ 5 mW cm^–2^, measured using a power meter. The light-induced surface potential changes correspond to surface photovoltage.

**Scanning Electron Microscopy.** SEM imaging was performed after Au/Pd sputtering of the sample on carbon sample holders in a Zeiss LEO 1550-Gemini system (acceleration voltage: 3 to 10 kV).

**Transmission Electron Microscopy and Electron Energy Loss Spectroscopy.** The measurements were acquired using a double-Cs-corrected Jeol JEMARM200F microscope, equipped with a cold field emission gun, a Gatan GIF Quantum detector, and a JED-2300 energy-dispersive X-ray detector. The acceleration voltage was typically set to 80 kV.

**X-Ray Photoelectron Spectroscopy.** XPS measurements were performed using CISSY equipment with a SPECS XR 50 X-ray gun Mg Kα radiation (1254.6 eV) and Combined Lens Analyzer Module (CLAM).

**Fluorescence Spectroscopy.** Photoluminescence spectra were recorded by Horiba FluoroMax-4 equipped with a thin film sample holder set at 60° with respect to the excitation, integration time 0.2 to 1 s, and slits apertures 1.

**Ion number calculation.** Some variables used in this work are as following:

Current density: J=9 μA/cm^2^

Active area (membrane area): S=πr²=π×0.25×0.25=0.0625 cm^2^

Channel density: D=5×10^9^ /cm^2^

Ion number per channel: X_Cation_=X_Anion_=X_Current_/2

e=1.602×10^-19^ C

Time: t=1s

The current density can be expressed by:

J=I/S=Q/S×t= D×S×e×X_Current_ / S×t

Therefore, the ion number per channel per second in this work is:

X_Cation_=X_Anion_=X_Current_/2= J ×S×t / 2×D×S×e= J / 2×D×e ≈ 5500 per channel per second


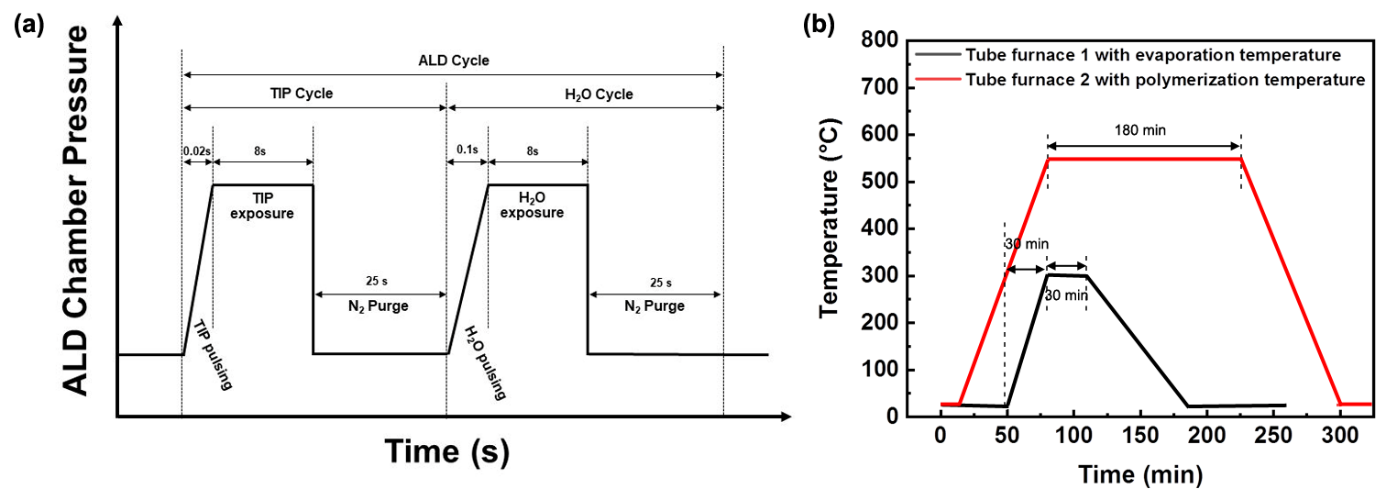


**Figure S1.** **a**, The temperature and time set for the fabrication of TiO_2_ layers. **b**, The temperature set in accordance with the time variation in two tube furnaces of CVD.


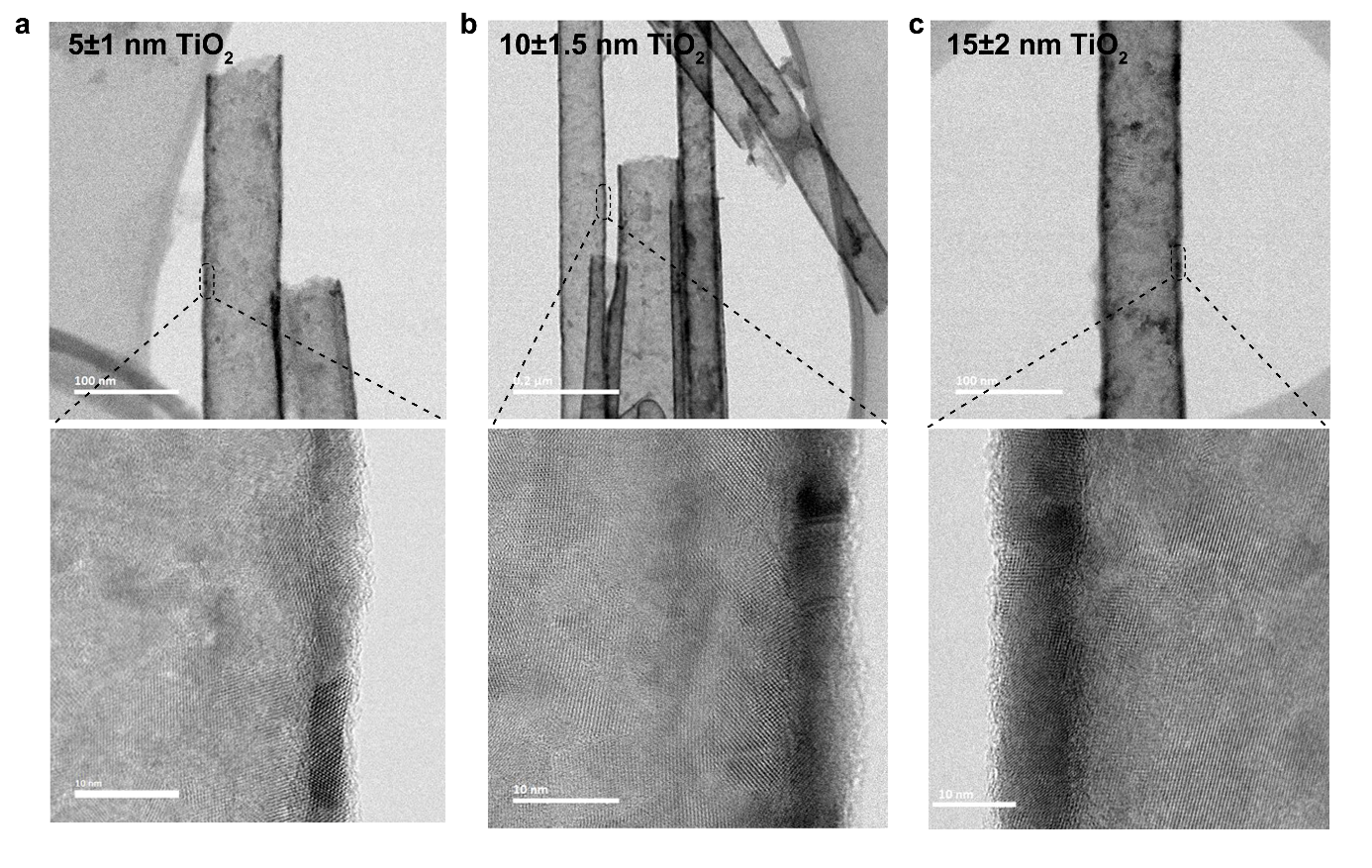


**Figure S2.** SEM images of TiO_2_ nanotube and enlarged wall with average thickness of 5 nm (**a**), 10 nm (**b**), and 15 nm (**c**).


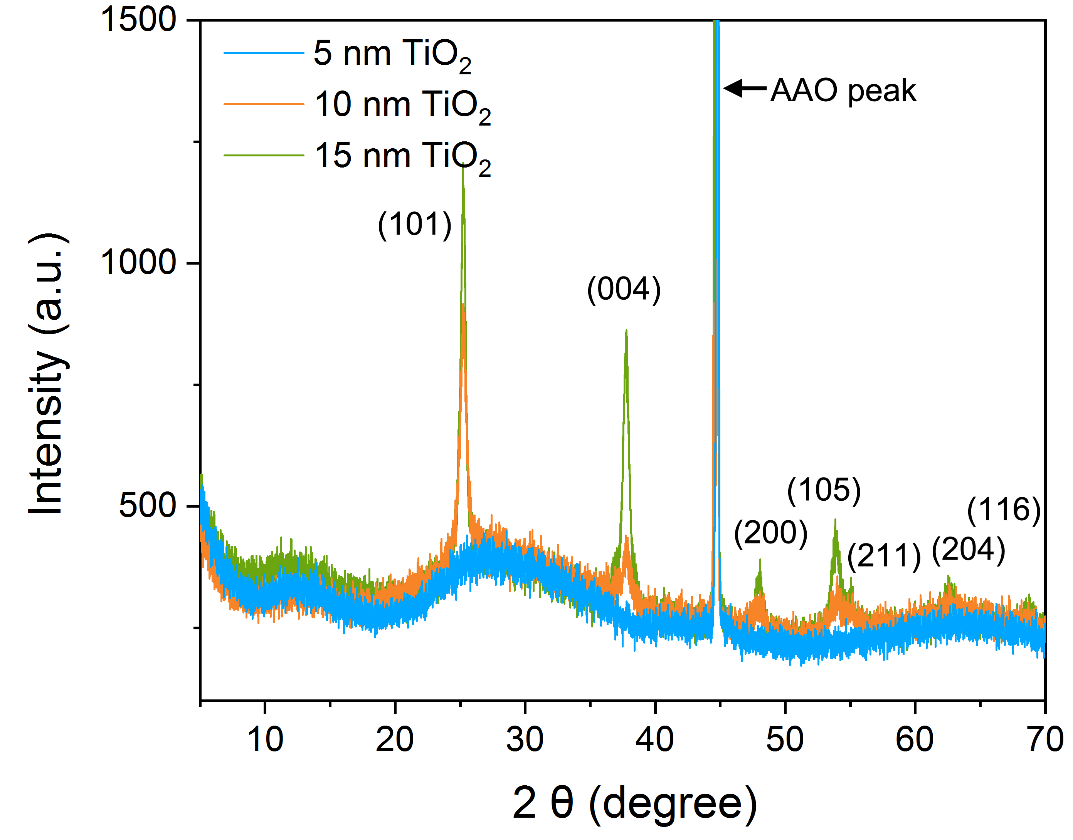


**Figure S3.** XRD of TiO_2_ nanotubes with average thickness of 5 nm (Blue), 10 nm (Orange), and 15 nm (Green). The signal increases gradually with the wall thickness. Meanwhile, the typical peaks indicate that the TiO_2_ nanotubes changed from amorphous phase to anatase phase.


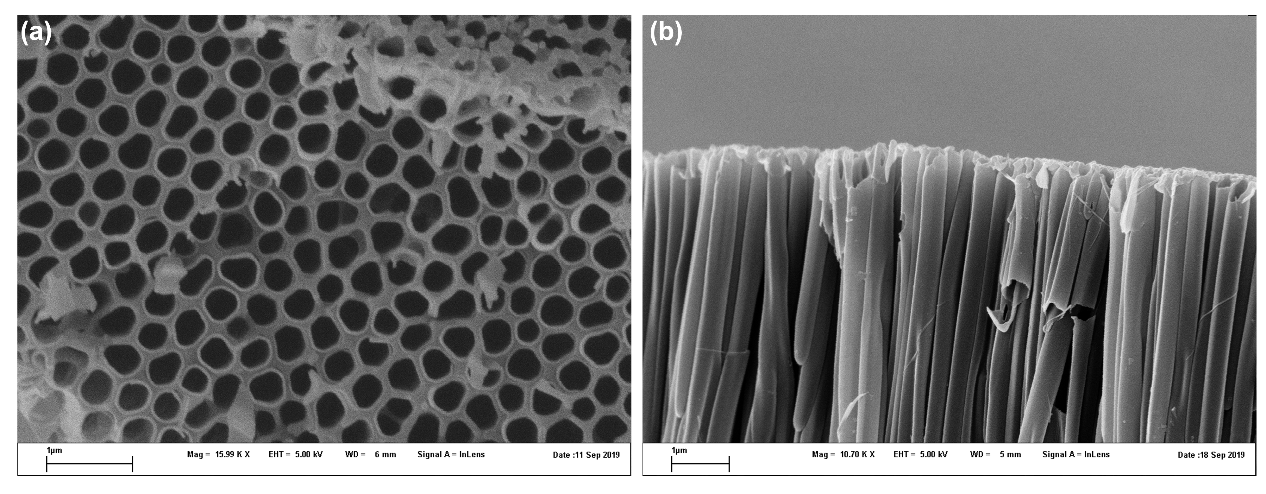


**Figure S4.** **a,** SEM image of the C_3_N_4_ covered AAO membrane. **b**, The C_3_N_4_ nanotube membrane with wall thickness of 10 nm after removing AAO substrate.


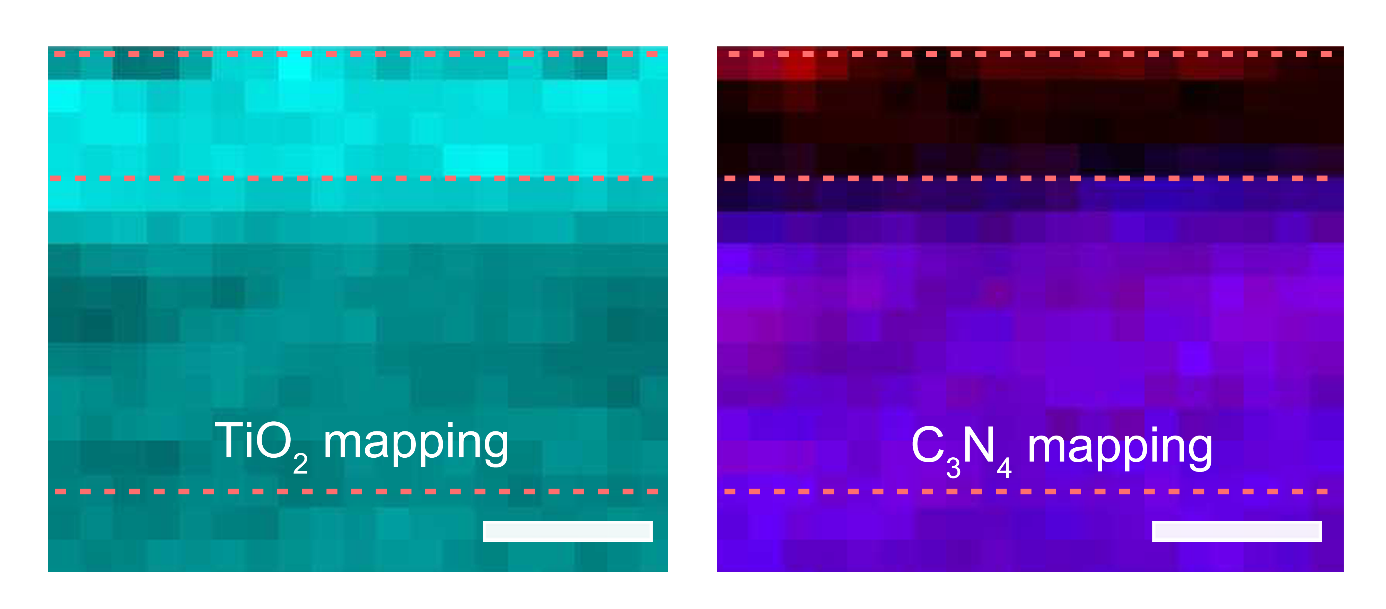


**Figure S5.** The TiO_2_ and C_3_N_4_ mappings by overlapping the elements distribution, scale bar 5 nm.


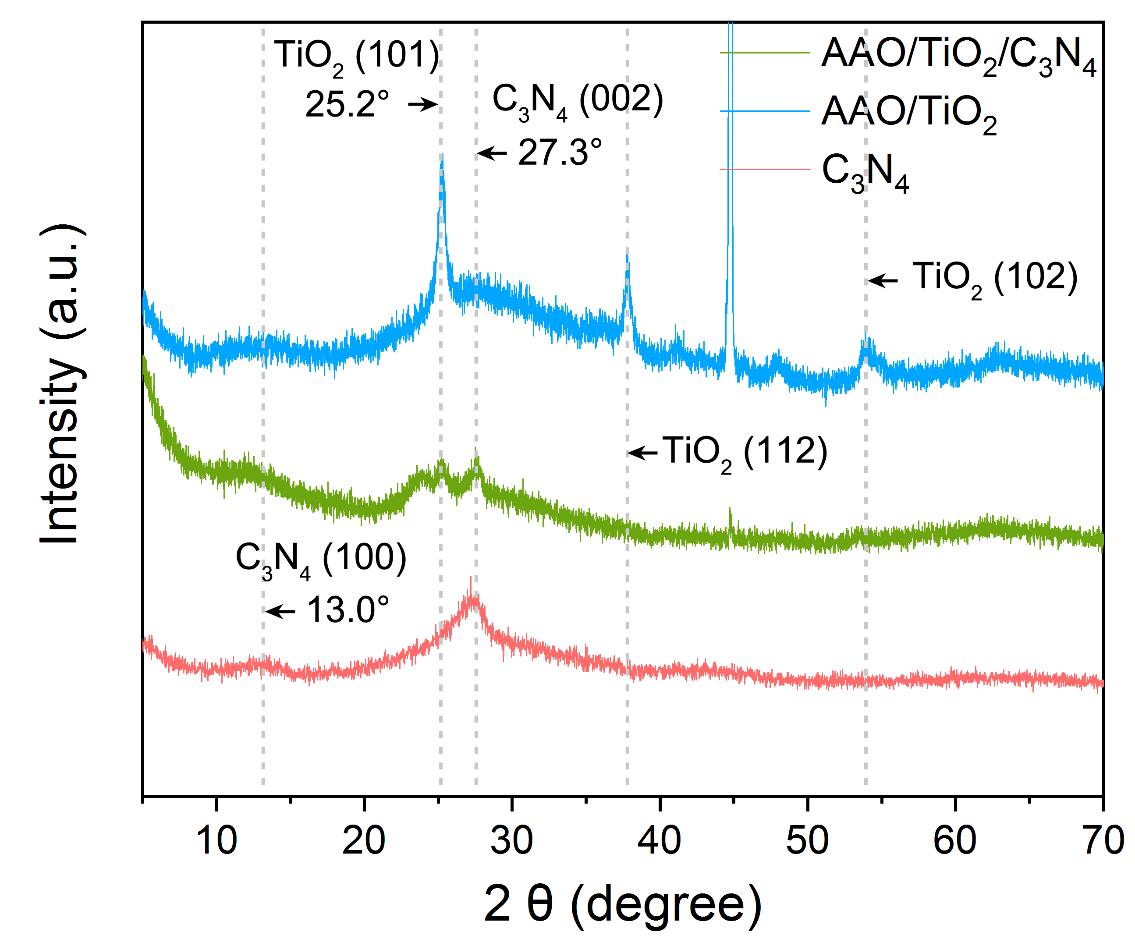


**Figure S6.** XRD of TiO_2_, C_3_N_4_, and TiO_2_/C_3_N_4_ nanotube membranes.





**Figure S7.** FT-IR of bared AAO, TiO_2_, C_3_N_4_, and TiO_2_/C_3_N_4_ nanotube membranes.


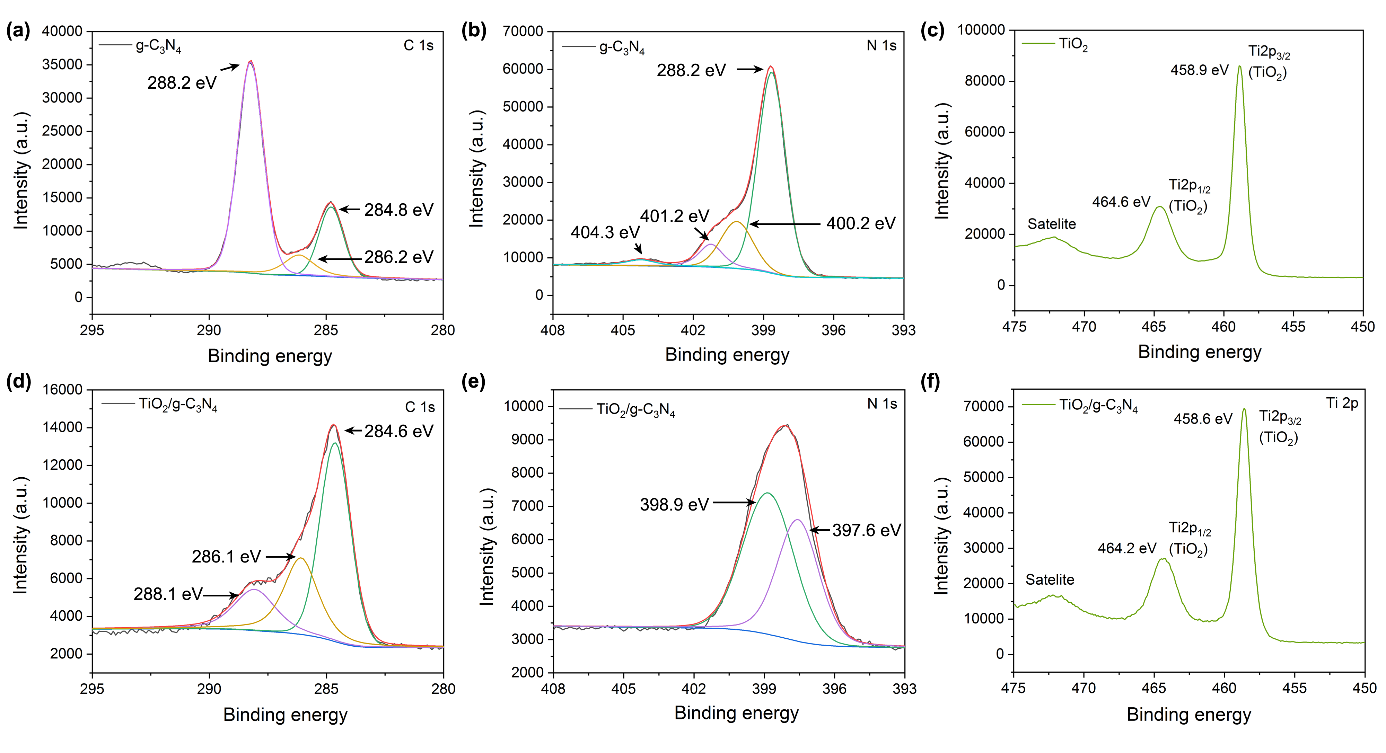


**Figure S8.** XPS of C_3_N_4_ and TiO_2_/C_3_N_4_ nanotube membrane. (a) C 1s of pure C_3_N_4_ nanotube membrane; (b) N 1s of pure C_3_N_4_ nanotube membrane; (c) Ti 2p of pure TiO_2_ nanotube membrane; (d) C 1s of TiO_2_/C_3_N_4_ nanotube membrane; (e) N 1s of TiO_2_/C_3_N_4_ nanotube membrane; (f) Ti 2p of TiO_2_/C_3_N_4_ nanotube membrane. The C 1s and N 1s spectra between the pure C_3_N_4_ nanotube and TiO_2_/C_3_N_4_ nanotube are different and quantitatively confirm the generation of a TiO_2_/C_3_N_4_ heterojunction. For C 1s peaks, the high energy peak 288.2 eV of pure C_3_N_4_ nanotube decreased to 288.1 eV of TiO_2_/C_3_N_4_ nanotube and the peak area decreased obviously, which means electrons transformation from TiO_2_ to C_3_N_4_ in the heterojunction. The N 1s spectrum of TiO_2_/C_3_N_4_ nanotube however only has two low energy peaks of 397.6 eV and 398.9 eV compared to pure C_3_N_4_ nanotube which has four peaks. The disappearance of peaks in 404.3 eV and 401.2 eV quantifies also electron transfer from TiO_2_ to C_3_N_4_ to the most positive nitrogen atoms.

**
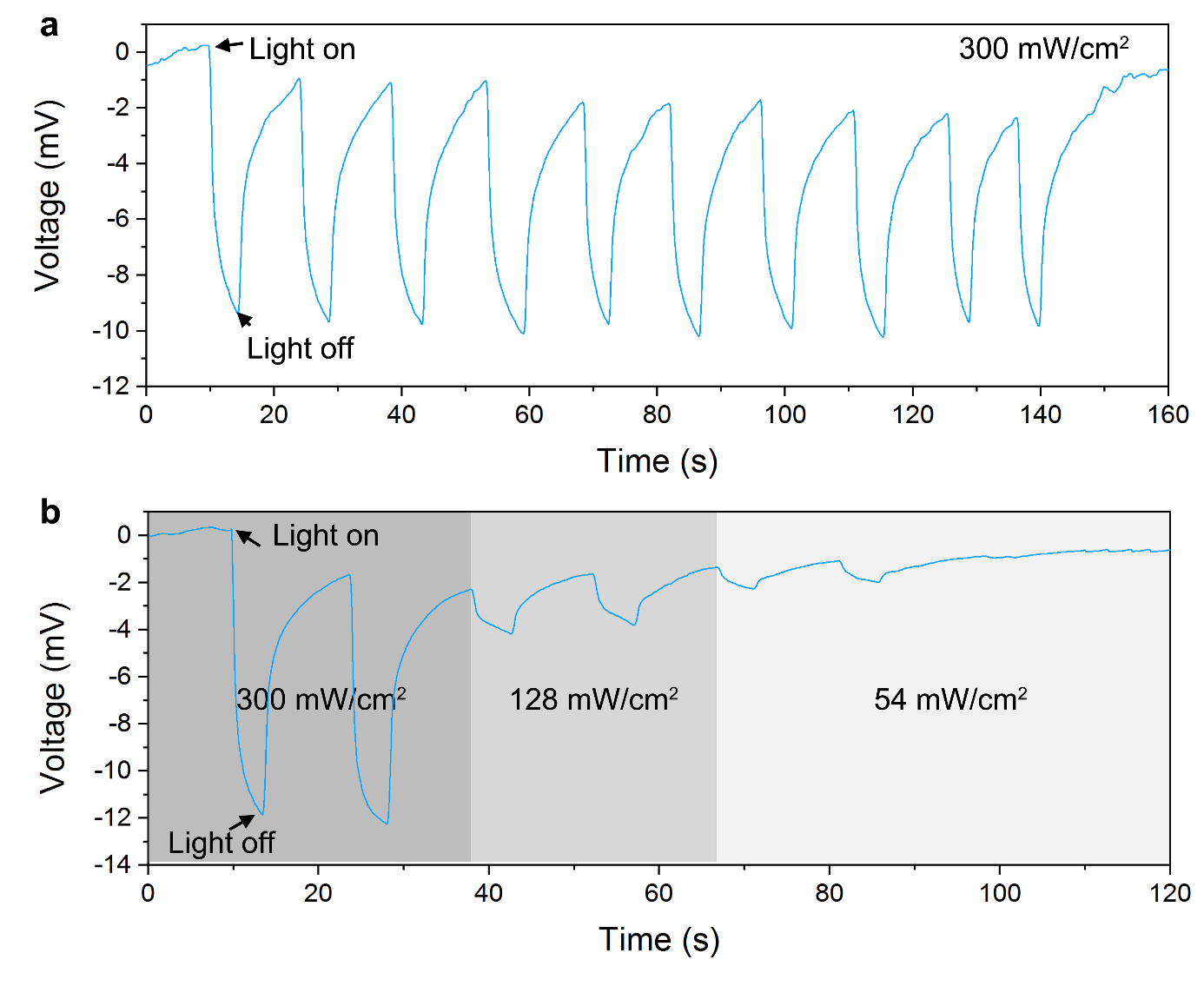
Figure S9.** **a**, Measured cyclic constant open-circuit voltage with the alternating illumination at 0.1 M KCl concentration. **b**, Open-circuit voltage as a function of light density of 54 mW/cm^2^, 128 mW/cm^2^ and 300 mW/cm^2^.


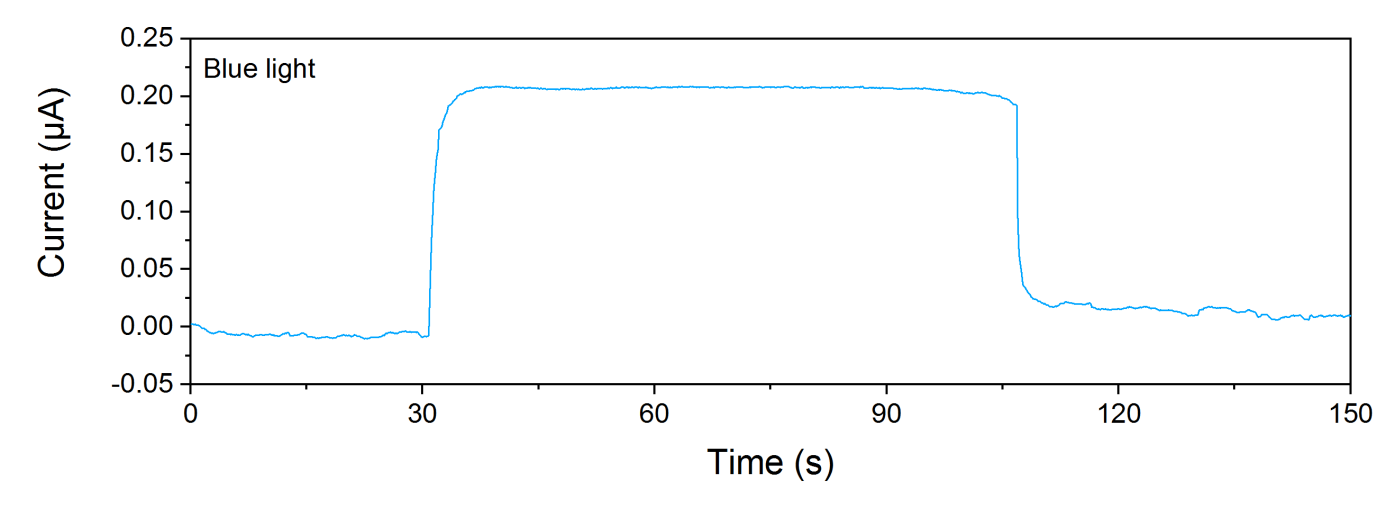


**Figure S10.** Measured zero-volt current after 100 s light irradiation, which indicates the stability of this system.


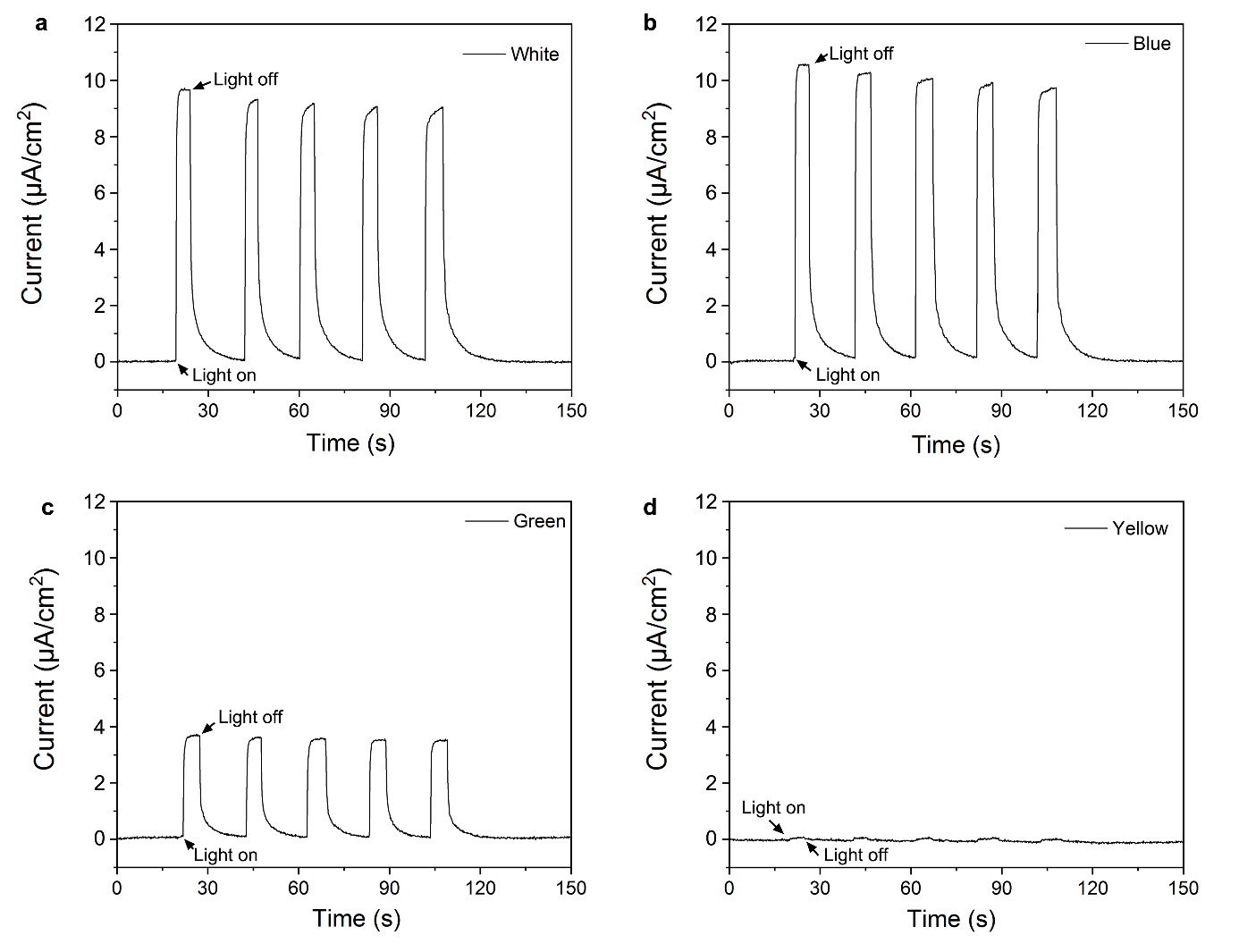


**Figure S11.** Measured zero-volt current by different light source (while, blue, green, and yellow) with same power density (300 mW/cm^2^).





**Figure S12.** UV-vis absorption of C_3_N_4_ nanotube membrane.


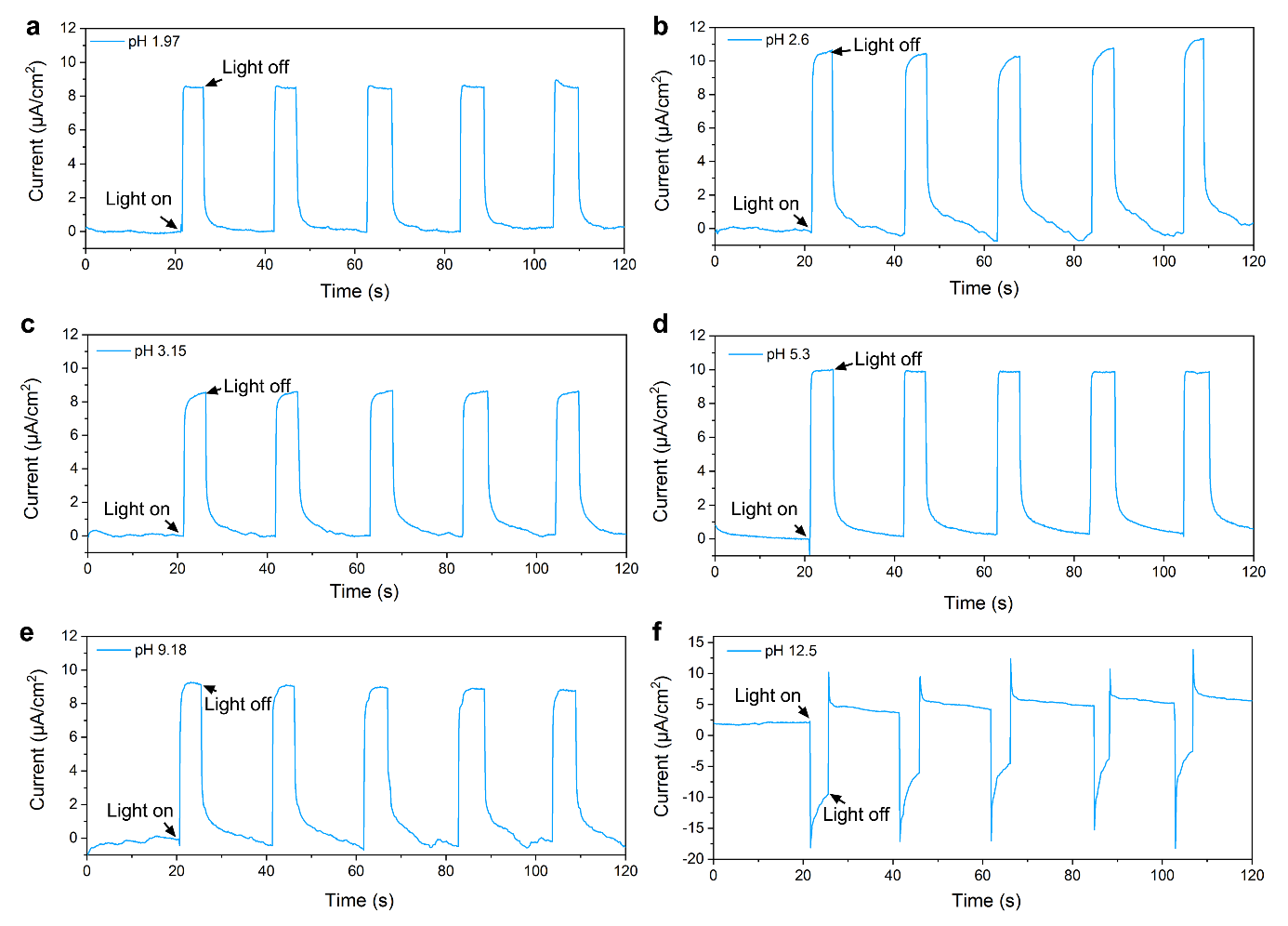


**Figure S13.** Measured zero-volt current in different pH value. The thickness of TiO_2_ used here is about 5 nm. The ionic current is stable with pH value from 1.97 to 9.18 (**a**-**e**), while it is abnormal in high pH value 12.5 (**f**). Two possible reasons should be responsible for the different ionic current direction in strong base condition (12.5). One is the high negative charge density in strong alkaline solution. Another one is that the band gap of C_3_N_4_ changed in strong alkaline solution, which then changed the directions of separated holes and electrons in the semiconductor heterojunction. By this way, the ions will move in a different direction.


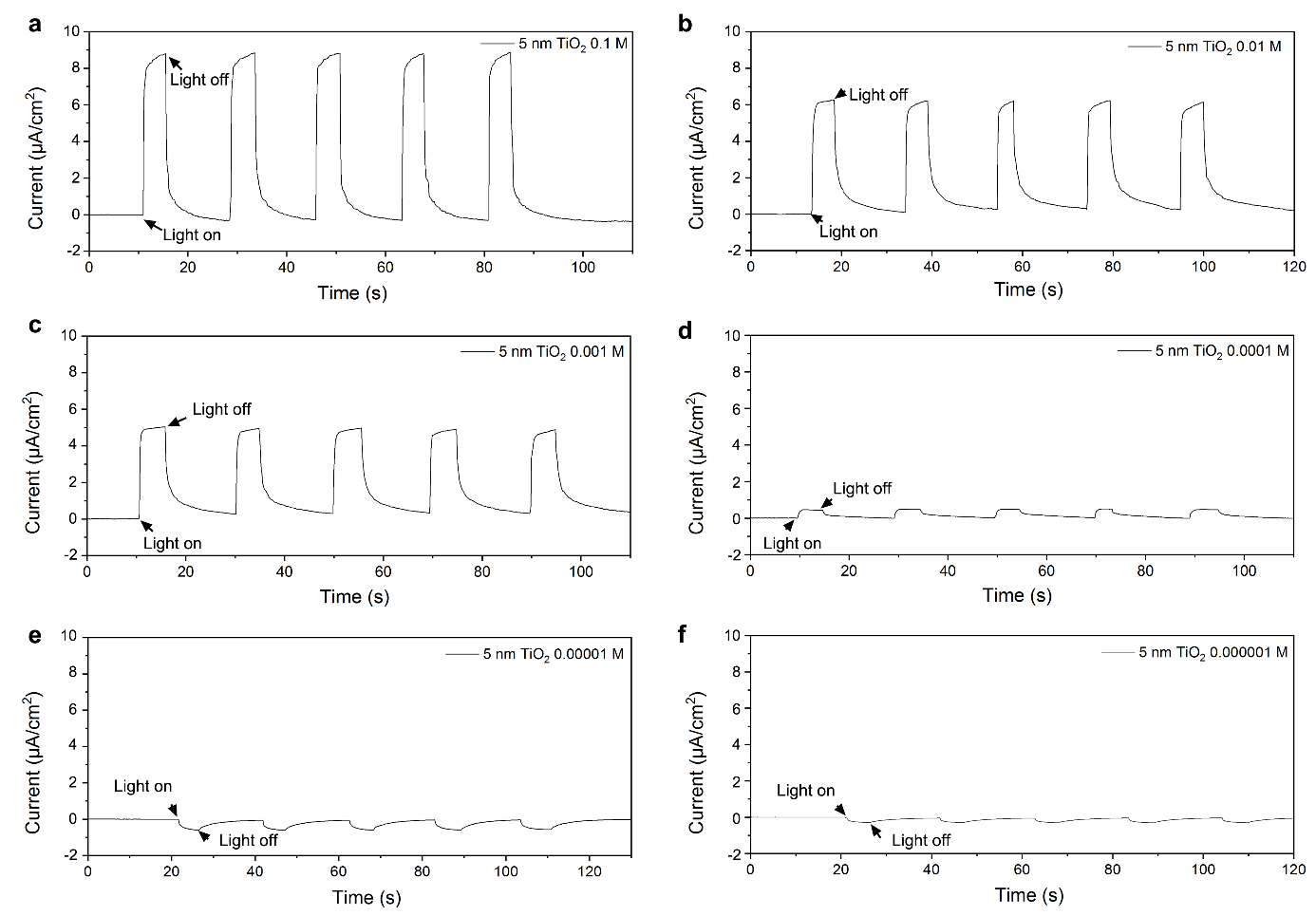


**Figure S14.** Measured zero-volt current in various electrolyte concentration. In generally, the ionic current decrease gradually with concentration. The thickness of TiO_2_ used here is about 5 nm. It is easy to understand that the ionic current decreases gradually with the concentration, while it is anomalous the ionic current changed its direction under 0.00001 M (e) and 0.000001 M (f). One possible reason is that light irradiation in this work still has weak effect to the Ag/AgCl electrode in spite of the protection function. Under low concentration, this effect will be more apparent. Yet, this also gave an indirect evidence that the photo-driven ionic current is resulting from semiconductor heterojunction effect.


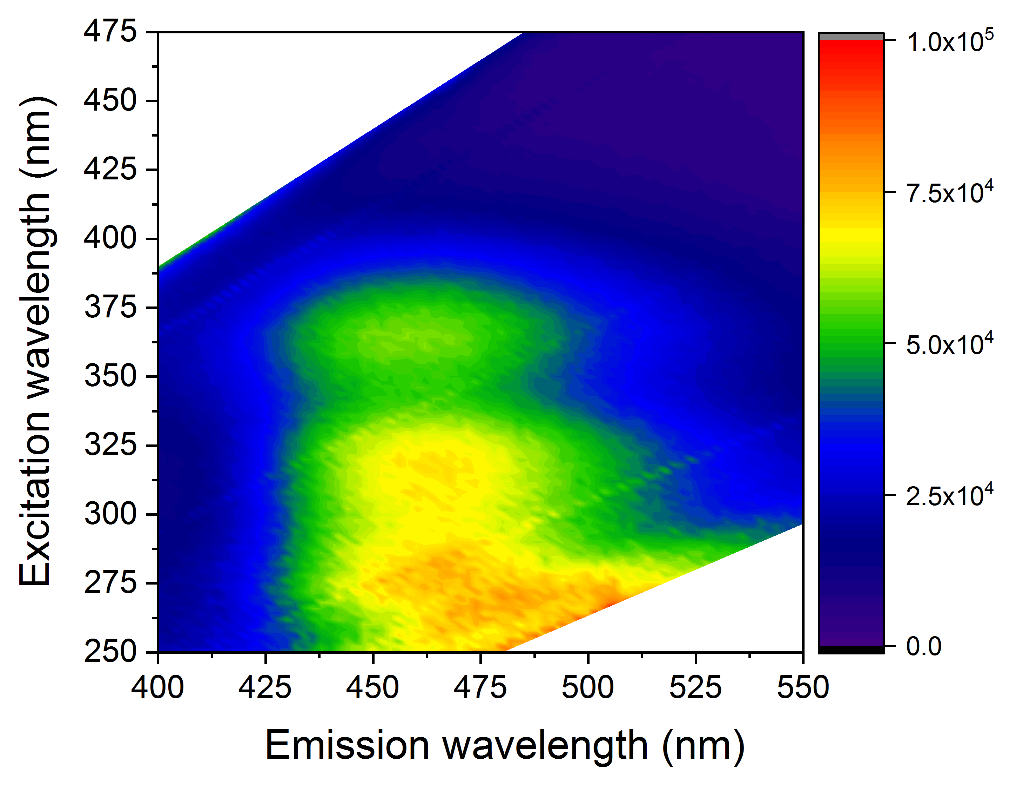


**Figure S15.** Fluorescence mapping of TiO_2_/C_3_N_4_ nanotube membrane with small scale bar (0-1.0 × 10^5^) while the scale bar in Fig.3d is 0-5 × 10^6^.


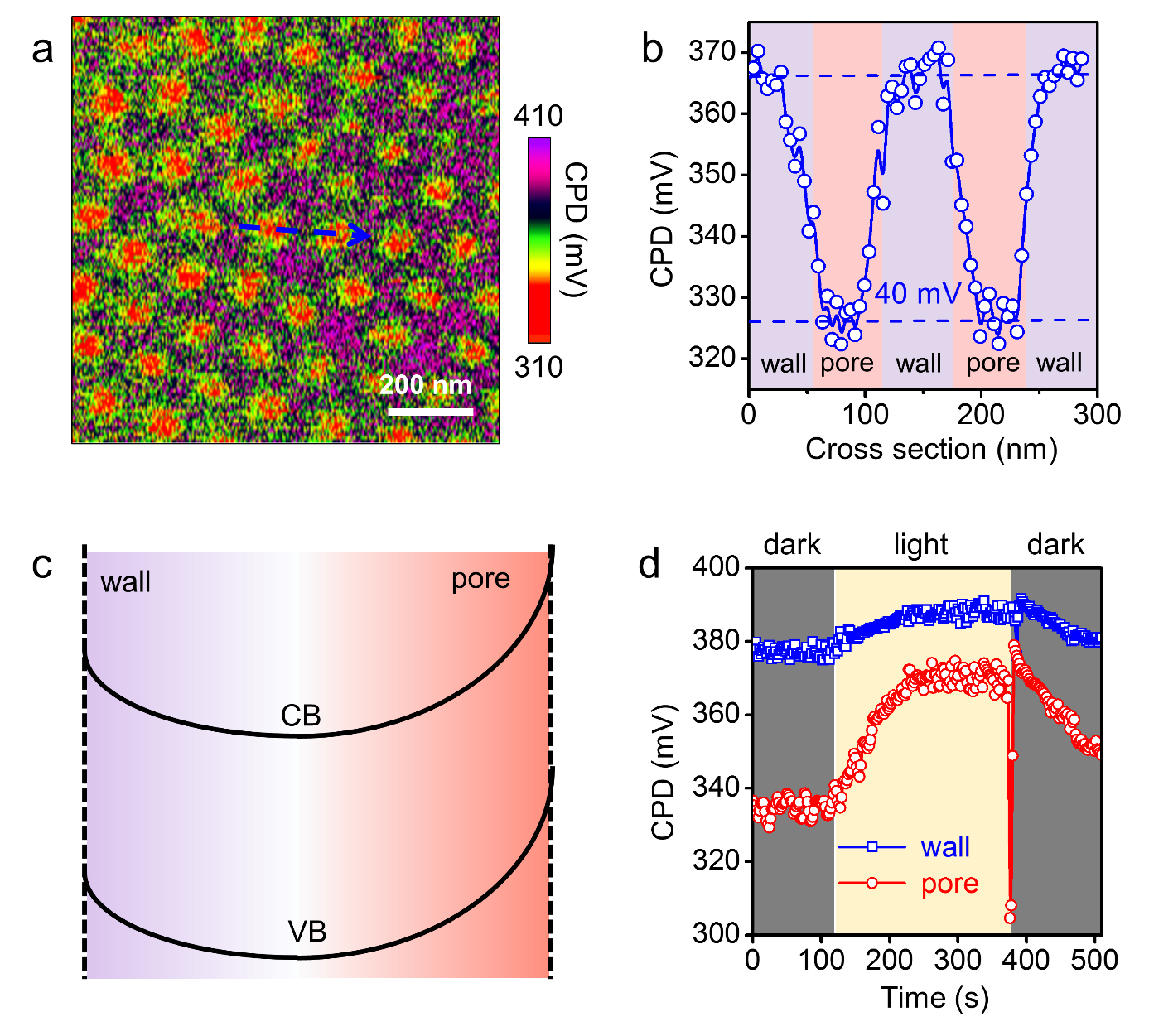


**Figure S16.** **a**, KPFM image of C_3_N_4_ nanotube membrane. **b**, CPD distributions along the dashed line in **a**. **c**, Deduced band diagram with wall area and pore area of C_3_N_4_ nanotube membrane on the basis of the measured CPD values. **d**, CPD evolution with light on and off in pore area and wall area of C_3_N_4_ nanotube membrane. The increased surface potential with illumination coincides with the upward band bending. The larger increase of surface potential under illumination (so-called surface photovoltage) in pore area agrees well with the larger band bending of pore area deduced by surface potential values.


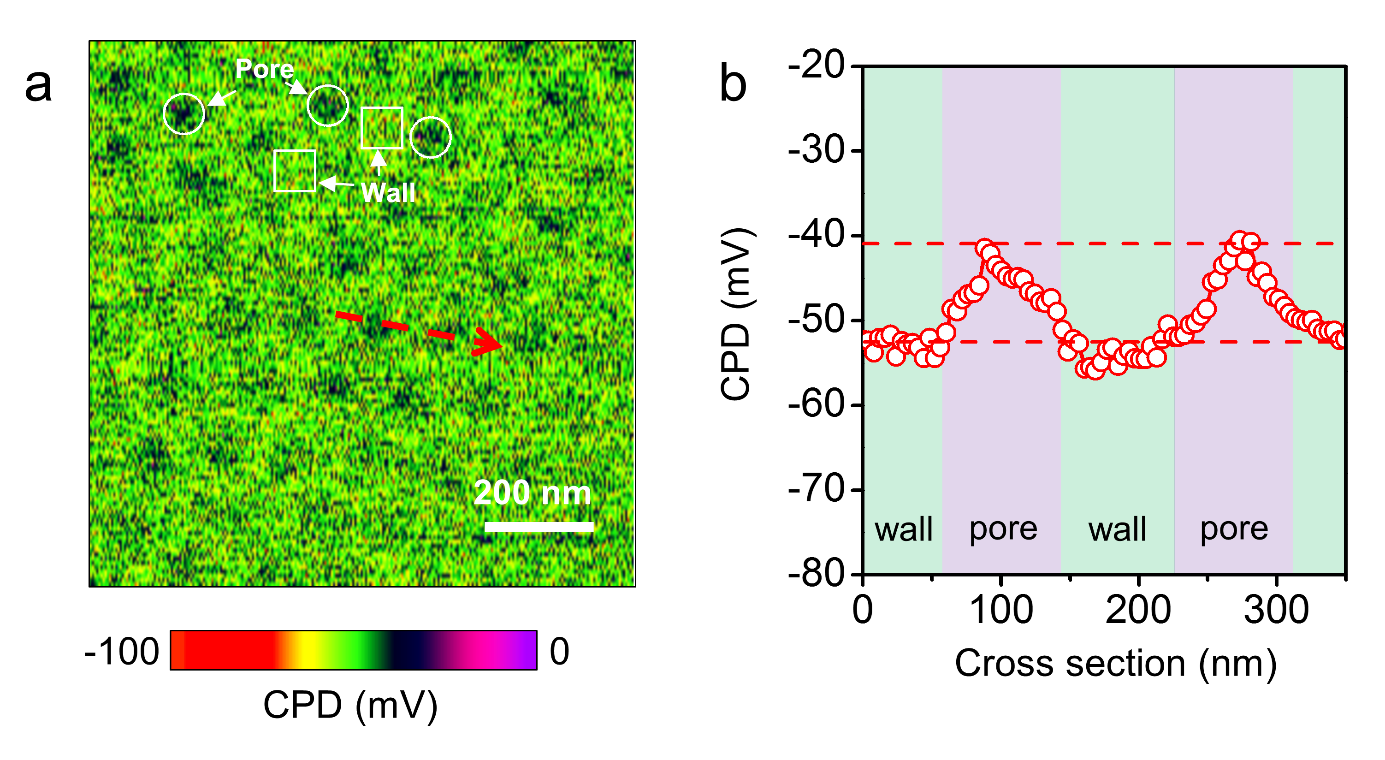


**Figure S17.** (**a**) KPFM image of TiO_2_/C_3_N_4_ nanotube membrane. The CPD scale is same with that in Figure S16a. Comparing two images indicates that the intrinsic potential difference between pore area and wall area strikingly decreases owing to the formation of heterojunction between TiO_2_ and C_3_N_4_. (**b**) CPD distributions along the dashed line in a. The CPD distributions show that the CPD of pore area is larger than that of wall area, the trend of which is opposite to the CPD distributions in Figure S15a, where the CPD of wall area is larger.

**Reference**

1. Giusto P, Cruz D and Heil T *et al.* Shine Bright Like a Diamond: New Light on an Old Polymeric Semiconductor. *Adv Mater* 2020; **32:** 1908140.

2. Chen L, Yan R and Oschatz M *et al.* Ultrathin 2D Graphitic Carbon Nitride on Metal Films: Underpotential Sodium Deposition in Adlayers for Sodium‐Ion Batteries. *Angew Chem Int Ed* 2020; **59**: 9067-9073.
